# Expansion of the Sahara Desert and shrinking of frozen land of the Arctic

**DOI:** 10.1038/s41598-020-61085-0

**Published:** 2020-03-05

**Authors:** Ye Liu, Yongkang Xue

**Affiliations:** University of California Los Angeles (UCLA), Los Angeles, CA USA

**Keywords:** Climate-change adaptation, Climate-change impacts, Climate-change mitigation, Environmental social sciences, Climate change

## Abstract

Expansion of the Sahara Desert (SD) and greening of the Arctic tundra-glacier region (ArcTG) have been hot subjects under extensive investigations. However, quantitative and comprehensive assessments of the landform changes in these regions are lacking. Here we use both observations and climate-ecosystem models to quantify/project changes in the extents and boundaries of the SD and ArcTG based on climate and vegetation indices. It is found that, based on observed climate indices, the SD expands 8% and the ArcTG shrinks 16% during 1950–2015, respectively. SD southern boundaries advance 100 km southward, and ArcTG boundaries are displaced about 50 km poleward in 1950–2015. The simulated trends based on climate and vegetation indices show consistent results with some differences probably due to missing anthropogenic forcing and two-way vegetation-climate feedback effect in simulations. The projected climate and vegetation indices show these trends will continue in 2015–2050.

## Introduction

Global climate change has extensively modified landforms and terrestrial ecosystems in many parts of the world during past decades^1,2^. Expansion of the Sahara Desert (SD) and greening of the Arctic tundra-glacier region (ArcTG) have profound societal and economic consequences and affected the regional and global climate^3–5^. They have been hot subjects under extensive investigations^1,2,5–10^.

The severe West African drought and land-use changes there in the 1970s-1980s caused land degradation and desert expansion, and deteriorated the food and water security in Sahelian countries^11,12^. The SD expansion has been used by the United Nations and countries/organizations as an indication for action and is a hot topic under debate^2,6–8,10^. The vegetation indicator, such as the normalized difference vegetation index (NDVI), has been used to identify the location of SD southern boundary^7^. It is reported that the interannual fluctuations of SD southern boundary based on NDVI similar to that based on isohyet definition in 1980–1997^13^. Thomas & Nigam^2^ used precipitation as an indicator to define SD boundary and reported that the SD expands 10% during the 20th century. NDVI is calculated as the ratio between reflectance of a red band (RED) and a near-infrared band (NIR), NDVI = (NIR-RED)/(NIR + RED). It is a measure of chlorophyll abundance and energy absorption. Therefore, NDVI is just a qualitative measurement of vegetation conditions. While, leaf area index (LAI) provides a plant property measurement for plant density and growth, LAI is more accurate in quantifying surface vegetation condition and landform change. Therefore, LAI is used to identify the SD boundary, which can more realistically distinguish bare-ground and vegetated area and better represents SD landform change. Furthermore, although precipitation dominates the dryland ecosystem, the warming-induced high potential evaporation has additional impacts on regional drying^14^. Heat stress, particularly after the 1980s, is found to harm the recovery of the Sahelian ecosystem^15^. Temperature is considered as another important indicator to assess dryland conditions^16^. The Köppen-Trewartha climate (KTC) index, which is associated with both precipitation and temperature and their seasonality, provides a globally coherent metric to quantify the landform change^17^. This index also relates climate variables to surface land cover types when it was designed^18^. The distribution of the world’s major ecosystems and the KTC zones has shown a high degree of correspondence^18^. In this study, we use both LAI and KTC index to define the SD boundary to investigate current and future SD areal extent and boundary changes.

Another region, the Arctic, that is investigated in this study is warming faster than the global average (“Arctic amplification”)^19^, resulting in changes in tundra ecosystem^9,20–23^. Evidence from several circumarctic treeline sites shows a clear invasion of tree and shrub into previous tundra area^9^, suggesting a decrease in the area of ArcTG. The northward shift of treeline would decrease high-latitude albedo and provide positive feedback, further enhancing global warming^24^. National Academies of Science, Engineering, & Medicine^5^ have reported recent substantial vegetation condition changes (greening and browning) in the Arctic region, and the implication of such vegetation changes. The northern treeline and summer temperature are used to define the boundary of ArcTG^25^. In fact, the treeline is nearly coincident with isotherm definitions over most Arctic land areas^26^. The KTC index (similar to previous thermal definition) is able to track Arctic tundra area shrinking. In this study, treeline and KTC index are both used as vegetation and climate indicators, respectively, to define the boundary of ArcTG and investigate their changes.

Global climate change has led to remarkable vegetation condition and landform change at the global scale. Simultaneous changes are taking place in many regions across the globe, especially Sahelian regions and the Arctic have received more attention. Thus far, published literature normally discussed the land condition changes in these two regions in separate articles, and most study use only precipitation for SD and temperature for ArcTG. For vegetation conditions, most studies focused on changes in NDVI and other vegetation indices^5,27^. The 1980s climate regime shift represented a major change in the Earth systems from the atmosphere, land to the ocean, which is identified by abrupt mean status shift and trend change in temperature, precipitation, sea surface pressure, terrestrial ecosystem conditions, and many other variables^15,28^. Therefore, we also assess the decadal variability in SD, in addition to identify one trend for the entire period as did in many other studies^2^.

In this study, we use satellite LAI and treeline products to derive observed vegetation index and gridded precipitation and temperature data to construct observed climate index. The National Centers for Environmental Prediction (NCEP) Climate Forecast System version-2 (CFSv2) coupled with the Simplified Simple Biosphere model version 2 (CFS/SSiB2), and coupled with a dynamic vegetation model (CFS/SSiB4), are used in this study. The dynamic vegetation model allows vegetation coverage, LAI, and relevant surface biophysical properties such as roughness length to interact with climate, while in CFS/SSiB2, these vegetation parameters are specified based on a vegetation table (see “Models and outputs” in Method for detail). The comparison between results from CFS/SSiB2 and CFS/SSiB4 allows to investigate the two-way vegetation-climate feedback in landform change.

Vegetation index directly reveals geographic boundaries of SD and ArcTG and their changes. The satellite based the vegetation index only covers the period after the 1980s when satellite data becomes available. Climate index has shown consistent results with that of vegetation index^6,7,29^, and has longer records. Therefore, the climate index is used to investigate long-term trend and decadal variability of the areal extent and boundary changes over SD and ArcTG in this study. The results from both climate index and vegetation index are cross-validated, and the possible causes for their difference are discussed. The areal extents derived from climate index will be denoted with a subscript of “OBS-Clim” for observation and “CFS/SSiB2-Clim” and “CFS/SSiB4-Clim” for CFS/SSiB2 and CFS/SSiB4 simulations, respectively. For the vegetation index, “Veg” will replace “Clim” accordingly. The statistics for their areal extents and changes are summarized in Table 1.

**Table 1:** The areal extents and their change rates for the Sahara Desert (SD), and the Arctic Tundra-Glacier (ArcTG) region, based on climate and vegetation indices from observations and model simulations.

Data Sources	Abbreviated Subscript	SD			ArcTG		
		Extent (10^6^ km^2^)	Rate (10^3^ km^2^/year)		Extent (10^6^ km^2^)	Rate (10^3^ km^2^/year)	
			1950–2015	2015–2050		1950–2015	2015–2050
**Climate Index**							
Observation	OBS-Clim	9.5	11.0*		5.7	−14.0*	
CFS/SSiB2	CFS/SSiB2-Clim	9.0	7.6	5.4	5.9	−5.0*	−16.0*
CFS/SSiB4	CFS/SSiB4-Clim	9.0	8.0*	6.6	5.8	−10.0*	−18.0*
**Vegetation Index**							
Observation	OBS-Veg	9.5					
CFS/SSiB4	CFS/SSiB4-Veg	9.6	8.0*	6.9	6.8	−13.0*	−17.0*

The extents are averaged over 1950–2015. The blank indicates no data cover this period. *Indicates the value with significant level at p < 0.01 (Mann-Kendall test).

## Results

### The sahara desert (SD)

**Figure 1:**
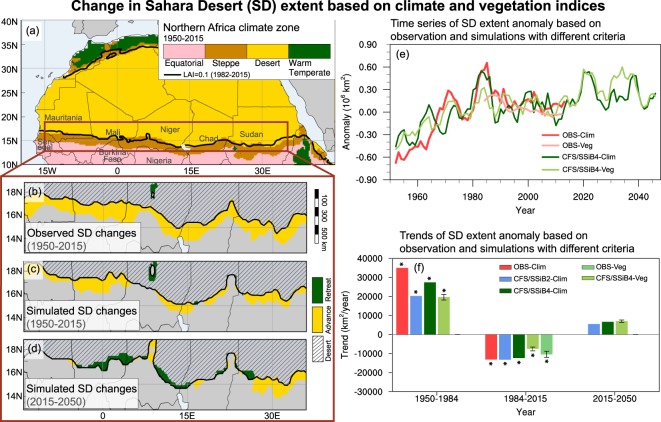
The Sahara Desert (SD) extent and boundary change based on climate and vegetation indices. (**a**) The Northern Africa climate zones averaged over 1950–2015. SD southern boundary changes based on climate index from (**b**) observation and (**c**) CFS/SSiB4 simulation during 1950–2015, and (**d**) CFS/SSiB4 simulation during 2015–2050. Observed and simulated (**e**) time series and (**f**) trend of SD extent defined by climate and vegetation indices. The error bars in (**f**) indicate one standard deviation due to the LAI based non-vegetation criterion range of 0.08–0.12 m^2^/m^2^. * in (**f**) indicates the value with significant level at p < 0.01 (Mann-Kendall test). Figure including maps in (**a–d**) are created by NCL (version 6.6.2, https://www.ncl.ucar.edu).

During 1950–2015, observed climate index shows that SD_OBS-Clim_ covers about 9.5 × 10^6^ km^2^ across North Africa (Fig. 1a and Table 1), within the range reported by Tucker *et al*.^6^. SD_OBS-Clim_ has a general expansion during 1950–2015, some 11,000 km^2^/year and increases 8% during 1950–2015, which is generally consistent with the previous studies^2^. The southern boundary of SD_OBS-Clim_ advances southward about 100 km from 1950 through 2015 (Fig. 1b). However, this general expansion is not constant in time. The Sahel areas experienced a dramatic change from wet conditions in the 1950s to much drier conditions in the 1980s, then partially recovered after the 1980s. A climate regime shift has been identified during the 1980s^15,28^. Different from previous SD studies, which only identify one trend for the entire study period, the year 1984, is identified in this study as turning points according to Eq. (9) to indicate the SD expansion-shrinking periods. Consistent with the climate shift, the SD has an expansion of 35,000 km^2^/year (p < 0.01, Mann-Kendall test) during 1950–1984, and a shrinking of 12,000 km^2^/year (p < 0.01) in 1984–2015 (Fig. 1f). The largest southward expansion occurs during 1950–1984, with the southern SD boundary expanding by 170 km, and a total 1,200,000 km^2^ expansion (about twice of the area of France).

The simulated climate indices properly reproduce SD extent and its changes during 1950–2015 (Table 1). The time series of SD_CFS/SSiB2-Clim_ and SD_CFS/SSiB4-Clim_ are well correlated with SD_OBS-Clim_ (Fig. 1e), with the temporal correlations being larger than 0.71 (p < 0.01, five-year running mean). The CFS models generate about 7600 km^2^/year (CFS/SSiB2, p = 0.02) and 8000 km^2^/year (CFS/SSiB4, p < 0.01) expansion from 1950 through 2015, accompanied by the expansion of southern boundaries by 70 km (CFS/SSiB4, Fig. 1c). Meanwhile, the models properly reproduce SD shrinking rate during 1984–2015. However, both CFS/SSiB2 and CFS/SSiB4 underestimate the expansion rate before 1984 by about 30%. In the Sahel, cropland and pastureland have expanded by 30% in the 1980s compared to that in the 1950s^12^ due to overgrazing, deforestation, and poor land management^8,10^. A multi-model experiment has demonstrated the land use and land cover change (LULCC) contribution to the drought during the 1980s, which should cause land degradation^12^. This anthropogenic effect is missing in this CFS simulation, which may lead to underestimation of the SD expansion rate during 1950–1984. Moreover, consistently fewer changes in the CFS/SSiB2 simulation compared with that in CFS/SSiB4 in SD and following ArcTG demonstrate the importance of two-way vegetation-climate feedback in landform change. The CFS models reproduce up to 70% of the observed expansion trend during 1950–1984 without consideration of LULCC in models. Meanwhile, during the SD shrinking period, while no remarkable LULCC occurred, CFS models are able to reproduce the observed shrinking trend. Therefore, the climate factors dominate SD changes compared to other effects, such as LULCC.

For the future projection through 2050 with the Representative Concentration Pathway (RCP) 4.5 scenario of the Intergovernmental Panel on Climate Change 5th Assessment Report (AR5), which only CFS is capable to conduct, the simulated climate indices show that with no LULCC the SD will further expand by about 6000 km^2^/year (p = 0.18 for CFS/SSiB2 and p = 0.15 for CFS/SSiB4). An asymmetrical boundary shift is projected, with about 40 km northward displacement in the western Sahel and 60 km southward displacement in the eastern Sahel (Fig. 1d). In the future projection, the Sahel temperature is projected to be about 1.8 °C warmer than the mean of 1986–2015. Despite the projected increase in precipitation in the mid-21st century, the warming-induced high evaporation dominates and makes the area drier and yields an SD expansion. The heat stress on Sahel ecosystem is well represented in KTC and has important implication for the future projection. Meanwhile, the projected heterogeneous precipitation anomaly distributions result in different desertification risks for various Sahelian countries.

Different from previous similar studies, in this study, we have also used vegetation indices derived from observation and a coupled climate-ecosystem model to assess the SD extend and its change, which provides a more clear geographic definition and can be used to cross-validate the results from the climate index. This ecosystem model has been extensively evaluated for its performance on north American and global ecosystem variability and trend^15,30^. We employ a range of 0.08–0.12 m^2^/m^2^ as the non-vegetation criterion to calculate the SD extent and its deviation with the assigned LAI range. The observed and simulated mean geographic SD extents (SD_OBS-Veg_ and SD_SSiB4-Veg_) based on this range are 9.5 × 10^6^ km^2^ and 9.6 × 10^6^ km^2^, respectively, with boundaries nearly coincident with those based on their corresponding climate indices (Fig. 1a).

The SD_OBS-Veg_ starts in the 1980s when the satellite data are available and records the SD recovery period. During 1984–2015, the SD_OBS-Veg_ shows a reduction of 10,000 ± 2000 km^2^/year (p < 0.01), close to the change based on SD_OBS-Clim_ (12,000 km^2^/year, Fig. 1e,f). The simulated SD_CFS/SSiB4-Veg_ is about the same as the climate index with 8000 ± 800 km^2^/year (p < 0.01) expansion during 1950–2015. During 2015–2050, the SD_CFS/SSiB4-Veg_ has projected a 6900 ± 600 km^2^/year (p = 0.14) expansion, close to that derived from climate index. In addition, the time series of SD_CFS/SSiB4-Veg_ is also consistent with SD_CFS/SSiB4-Clim_ with a correlation coefficient of 0.73 (p < 0.01) (Fig. 1e,f) for the whole period of 1950–2050.

The southern boundary of SD_CFS/SSiB4-Veg_ expands 90 km southward during 1950–2015 and will advance 40 km further southward in the eastern Sahel during 2015–2050. In the western Sahel, no significant change is projected during 2015–2050, different from the projection based on climate index. The CFS/SSiB2 uses specified LAI. As such, no assessment can be made based on the vegetation index. With two definitions, we cross-evaluate the uncertainty in assessing/project SD expansion due to two different definitions and show they are generally consistent. Some discrepancies are likely due to errors in satellite-derived LAI and simulated climate and vegetation variables over the sparse vegetation area^31^.

### The arctic

**Figure 2:**
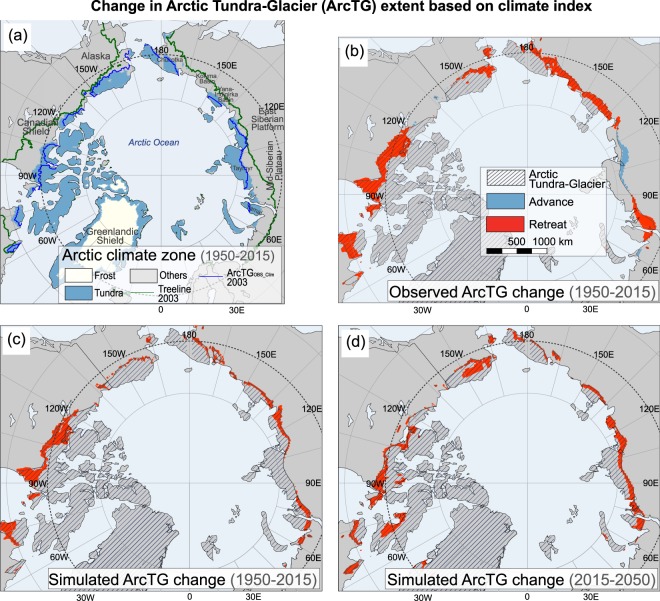
The Arctic Tundra-Glacier (ArcTG) extent and boundary change based on climate index. (**a**) The Arctic climate zones averaged over 1950–2015. ArcTG boundary changes based on climate index from (**b**) observation and (**c**) CFS/SSiB4 simulation during 1950–2015, and (**d**) CFS/SSiB4 simulation during 2015–2050. Figures including maps in (**a–d**) are created by NCL (version 6.6.2, https://www.ncl.ucar.edu).

**Figure 3:**
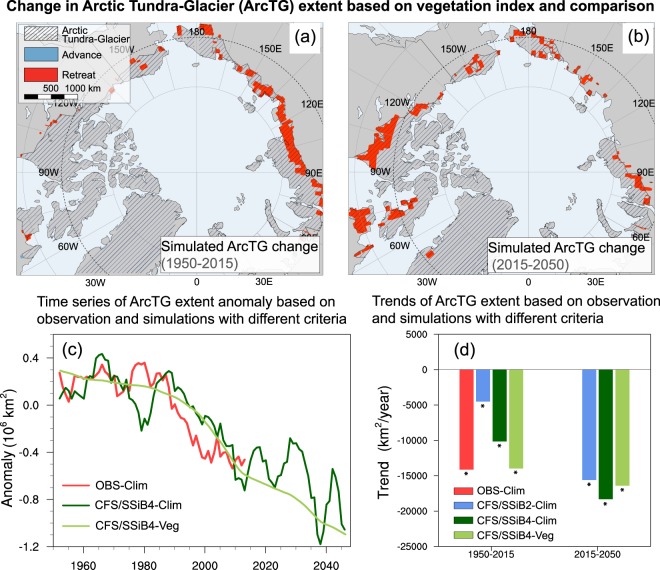
The Arctic Tundra-Glacier (ArcTG) extent and boundary change based on vegetation index and comparison. Changes of ArcTG based on CFS/SSiB4 simulated vegetation index during (**a**) 1950–2015 and (**b**) 2015–2050. Observed and simulated ArcTG extent (**c**) time series and (**d**) trends based on climate and vegetation indices. * in (**d**) indicates the value with significant level at p < 0.01 (Mann-Kendall test). Figures including maps in (**a**) and (**d**) are generated by NCL (version 6.6.2, https://www.ncl.ucar.edu).

The accelerated warming rate in the polar regions and intensive interactions between climate and vegetation, snow, and glacier have led to remarkable land condition changes in the ArcTG area in past decades (Lloyd *et al*., 2003; Swann *et al*., 2010; Schaefer *et al*., 2011; Pearson *et al*., 2013; Frost and Epstein, 2014), but reports on landform change at continental scale are lacking. The observed climate index shows that the average ArcTG_OBS-Clim_ covers 5.7 × 10^6^ km^2^ in 1950–2015 (Fig. 2a and Table 1) and is decreased at the rate of 14,000 km^2^/year (p < 0.01, 16% in total during this period, about the area of British Columbia, Canada) monotonically from 1950 through 2015 in response to global warming (Fig. 3c,d). The shrinking rate accelerates after the 1980s. The shrinking is accompanied by boundary retreat all over around the Arctic Circle (Fig. 2b): 60 km poleward in North America and 40 km poleward in Eurasia during 1950–2015.

The models generally reproduce the coverage of ArcTG and its changes based on climate index during 1950–2015 (Table 1). ArcTG_CFS/SSiB4-Clim_ diminishes at 10,000 km^2^/year (p < 0.01) during 1950–2015, with boundary retreats by 50 km in North America and 30 km in Eurasia (Fig. 2c), consistent with but lower than the ArcTG_OBS-Clim_. The CFS/SSiB2 with specified vegetation conditions, however, only reproduces one-third of the observed and CFS/SSiB4 simulated reduction rate (Fig. 3d). The lack of black carbon deposition and greenhouse gas emission in CFS may contribute to the discrepancies. In the Arctic, human-induced black carbon on snow is reported to accelerate the warming effect by enhancing surface radiative forcing^32^. The lack of greenhouse gas emission due to enhanced soil carbon respiration may also contribute to an underestimation of atmospheric warming^3,33^. The enhanced soil carbon respiration come from thawed permafrost, where microbial decay is increasing respiration CO_2_ and methane fluxes to the atmosphere. This in turn amplifies the rate of atmospheric warming and further accelerate permafrost degradation, resulting a positive permafrost carbon feedback. Meanwhile, the warming temperature and elevated atmospheric CO_2_ concentration cause an enrichment of shrubs and trees in the Arctic forest-tundra ecotone and produce positive feedbacks. In the future projection for 2015–2050, the simulated climate indices project about a 17,000 km^2^/year (p < 0.01) decrease in ArcTG extent, with 60 km retreat in North America and 40 km retreat in Eurasia by 2050 (Fig. 2d).

The observed vegetation index based on the products of CAVM treeline in the year of 2003 delineates the northernmost latitudes where tree species survive, which is defined as the geographical Arctic tundra and glacier southern boundary. The ArcTG_OBS-Veg_ (for the year 2003, green lines in Fig. 2a) covers 7.1 × 10^6^ km^2^, with a significantly larger area than ArcTG_OBS-Clim_ (for the year 2003, blue lines in Fig. 2a) in western Alaska, Canadian Shield, Taymyr Peninsula, and the Yamal Peninsula, where climate index seems to suggest trees are still able to survive. This is because the treeline dynamics are not only affected by the climate but also mediated by species-specific traits and environmental conditions such as permafrost thawing^34^, which deteriorates the local hydrological regime (such as active layer depth) and damages the root system that would prohibit tree establishment. These factors are not considered in the ArcTG_OBS-Clim_ and ArcTG_CFS/SSiB4-Clim_ and produce lower area extent estimation with these two indices compared to vegetation indices. We cannot assess either the long-term average of ArcTG_OBS-Veg_ extent or the advance rate using the CAVM treeline product since it is only for 2003. The treeline advance for the 20th century with various starting dates has been reported in a number of site measurements across the circumarctic forest-tundra ecotone^1,35,36^, indicating an Arctic shrinking in the past decades. The simulated ArcTG_CFS/SSiB4-Veg_ covers 6.8 × 10^6^ km^2^ for the period of 1950–2015, and covers 6.5 × 10^6^ km^2^ for the year 2003. The simulated ArcTG_CFS/SSiB4-Veg_ shrinking has consistency with the above-mentioned field measurements and shows a shrinking ArcTG during 1950–2015. The ArcTG_CFS/SSiB4-Veg_ boundary retreat, however, shows a different asymmetry in the North American and Eurasian continents compared to that indicated by the climate index. Although the Eurasian treeline shifts 50 km poleward, consistent with that of ArcTG_CFS/SSiB4-Clim_, but no significant change in the North American tree line is found for ArcTG_CFS/SSiB4-Veg_ (see Fig. 3a). The discrepancies between climate index and vegetation index in North America suggest that the shrinking of the ArcTG_CFS/SSiB4-clim_ there does not cause a significant treeline advance. The species-specific traits and local environmental conditions may also contribute to the treeline advance. In fact, the site observations in the Canadian Shield did not find the treeline advance in 20^th^ century^1^. In contrast, two sites in the Taymyr Peninsula, Siberia, had significant treeline advance^1^. These site measurements seem to be consistent with our simulation. Further assessments with more data are needed to reduce uncertainty. In the future projection, the treeline advance is predicted on both continents, with 60 km in North America and 30 km in Eurasia (Fig. 3b), resulting in a shrinking of the extent by 17,000 km^2^/year (p < 0.01, Table 1).

## Conclusions

In this paper, we assess landform changes in the Sahara-Desert and the Arctic tundra-glacier regions during 1950–2050, according to both climate index (with both precipitation and temperature) and vegetation index. In previous studies, only precipitation or NDVI was used to make an assessment in separate studies. We found that the area of SD expands 11,000 km^2^/year and 8,000/8,000 km^2^/year, during 1950–2015 based on the observed climate and CFS/SSiB4-simulated climate/vegetation index (no LULCC), respectively, and is projected to expand about 6600–6900 km^2^/year in 2015–2050, with southern boundary displace southward (Table 1). Extensive evaporation caused by warming temperature has contributed to the SD expansion in the future scenario. The climate factors dominate the variability of SD extent, while LULCC and two-way vegetation-climate feedback also play important roles in enhancing SD expansion.

The area of ArcTG reduces 14,000/10,000/13,000 km^2^/year during 1950–2015 based on observed climate/simulated climate/simulated vegetation indices (Table 1). The ArcTG will continue to expand about 18,000/17,000 km^2^/year during 2015–2050 based on simulated climate/vegetation indices. The shrinking is accompanied by boundary retreat across the circumarctic. CFS models tend to underestimate the ArcTG shrinking rate, mainly caused by missing anthropogenic process (such as black carbon in snow). The CFS simulation without dynamic vegetation substantially underestimates the shrinking rate, suggesting the two-way vegetation-climate interaction produces positive feedback and enhances the ArcTG shrinking. The discrepancies between the climate and vegetation indices reveal that the geographic changes are not only determined by the climate, but also affected by species-specific traits and local environmental conditions.

The land condition in these two regions have shown to have a substantial impact on climate, weather and ecosystems at continental and even, probably, global scales. We believe this article should stimulate more following scientific researches/debating on these subjects, which should provide useful information for economic and societal decisions with broad public interests.

## Methods and Data

### Vegetation index

The area with annual mean leaf area index (LAI) less than a threshold (0.1 m^2^/m^2^) in North Africa is defined as the geographic location of the Sahara Desert in this study. A range from 0.08–0.12 m^2^/m^2^ is used to assess the uncertainty of the threshold.

The treeline is defined as the edge of the habitat where tree species can grow, and thus it is regarded as tree fractional coverage equal to zero to its north. Treeline near the Arctic area is used to define the boundary of the geographic Arctic tundra-glacier area.

### Climate index

The KTC defines five temperature base groups (tropical, subtropical, temperate, boreal, and polar climates) and one precipitation base group. The threshold ($R$, in mm) distinguishing dry and wet climate is obtained according to the seasonal precipitation pattern and annual mean temperature as follows:

1 $$R=23T-6.4Pw+410,$$

where $T$ (in °C) is the annual mean temperature and $Pw$ (in %) is the percentage of annual precipitation occurring in the six coldest months. The dry climate is found where the annual precipitation ($P$, in mm) is less than $R$. Then, the dry climates are further divided into steppe ($P > 0.5R$) and desert ($P < 0.5R$) climates. However, the annual mean temperature in the transition zone between the Sahara Desert and the Sahel is about 26 °C, and less than 10% precipitation occurs in winter. Under these temperature and precipitation conditions Eq. (1) results in a 500-mm isohyet for deserts. It is significantly higher than the 200-mm isohyet, which is widely used to define the SD boundary^6,7^. Therefore, Eq. (1) is revised to

2 $$R=23T-6.4Pw-100,$$

and is used as the climate index to define the SD in North Africa (15 °W–35°E and 10 °N–30°N), which produces consistent isohyet with previous Sahara studies^6,7^.

The polar/tundra-frost climate in KTC is defined as the annual maximum of the monthly temperature less than 10 °C, which is used as an indicator to define the ArcTG zone in north of 55 °N in this study. This index is equivalent to the widely-used definition that the summer temperature is less than 10 °C^26^.

### Observations for surface temperature, precipitation, LAI, and treeline

Climatic Research Unit (CRU) time series (TS) provides gauge-based precipitation at 0.5° × 0.5° horizontal-grid and monthly temporal resolution^37^; version 3.24.01 was used. About 500 and 110 stations around the southern and northern boundaries of SD, respectively, had contributed to CRUTS precipitation assimilation at the beginning of the study period (1950). Global Historical Climatology Network/Climate Anomaly Monitoring System (GHCN_CAMS) gridded 2-m temperature over land at 0.5° × 0.5° resolution with monthly interval was also obtained^38^. These datasets are applied to calculate the climate index for the period 1950–2015 for SD (SD_OBS-Clim_) and ArcTG (ArcTG_OBS-Clim_). In 1950, there are about 40 and 35 stations located around the southern and northern boundaries of SD, respectively, while about 186 stations reported observed 2-m temperature in the north of 60 °N.

The Global Land Surface Satellite (GLASS) LAI was obtained to locate the non-vegetated area in North Africa. GLASS LAI was generated from AVHRR reflectance (1982–1999) and MODIS reflectance (2000–2012)^39^. The GLASS LAI provides observations at 8-day temporal resolution and 1-km spatial resolution for the period from 1982 to 2017. It is used to calculate the observed vegetation index for SD (SD_OBS-Veg_).

We also use the Circumpolar Arctic Vegetation Map (CAVM) treeline product^25^ to identify the geographic ArcTG (ArcTG_OBS-Veg_). This data set is only available for the year 2003.

### Models and outputs

The National Centers for Environmental Prediction (NCEP) Climate Forecast System version-2 (CFSv2)^40^ coupled with the Simplified Simple Biosphere model version-2 (CFS/SSiB2)^41–44^, and CFSv2 coupled with a dynamic vegetation model (CFS/SSiB4)^15,30,45–47^, are used in this study. The dynamic vegetation model allows vegetation coverage, LAI, and relevant surface biophysical properties such as roughness length to interact with climate, while in CFS/SSiB2, these vegetation parameters are specified based on a land cover map^48^ and a vegetation table^49^. The CFS has an interactive ocean component, the Modular Ocean Model version-4 (MOM4^50^), developed from the Geophysical Fluid Dynamics Laboratory (GFDL).

Two simulations are conducted using CFS/SSiB2 (without climate and ecosystem interaction) and CFS/SSiB4 (a dynamic vegetation process is included), respectively, integrated from 1949 through 2050, with T126 L64 spectral discretization (about 1° spatial resolution and 64 vertical levels). The ocean and atmospheric initial conditions are obtained from Lee *et al*.^43^ and the land initial conditions for CFS/SSiB4 are obtained from Liu *et al*.^15^. We first integrate the offline SSiB4 hundreds years to reach an equilibrium conditions, then using observed meteorological forcing to drive SSiB4 to obtain the vegetation conditions from 1949 to 2007. The 1949 conditions in Liu *et al*.^15^ is used as the CFS/SSiB4 initial conditions for this study. The simulations use atmospheric CO_2_ concentrations from the World Meteorological Organization (WMO) Global Atmospheric Watch (http://ds.data.jma.go.jp/gmd/wdcgg/) for the past and from a medium RCP scenario (RCP4.5) for the future and are updated once a year. The simulated temperature and precipitation from CFS/SSiB2 and CFS/SSiB4 are used to construct climate index, and the LAI and vegetation fraction from CFS/SSiB4 are used to calculate vegetation index. No vegetation index can be constructed from CFS/SSiB2 run. The difference between those two simulations implies the role of two-way vegetation-climate feedback on landform change. Model outputs are corrected with bias correction.

### Bias correction for the model outputs

In addition to observational data, model-simulated temperature, precipitation, and LAI are also used to determine the extents of the study areas. We conducted bias correction at each grid point as did in Bruyere *et al*.^51^ to minimize model systematic biases. The model-simulated variable (*Mod*′) is decomposed into a climatological mean component ($\overline{Mod}$) and a perturbation term (*Mod*′):

3 $$Mod=\overline{Mod}+Mod{\prime} ,$$

The observational data ($Obs$) is similarly decomposed into a climatological mean component ($\overline{Obs})$ and a perturbation term (*Obs*′)

4 $$Obs=\overline{Obs}+Obs{\prime} ,$$

The bias-corrected simulated variable (Mod^*^) is written as:

5 $$Mo{d}^{\ast }=\overline{Obs}+Mod{\prime} ,$$

### Endpoint method

Both the total area and boundaries are calculated for the Sahara Desert (SD) and the Arctic tundra-glacier (ArcTG) in this study. For convenience, we use the SD as an example in the following presentation. To do so, the total SD area for each year is obtained by taking an area sum after weighting each grid-cell area classified as SD multiplied by the cosine of its latitude. The SD time series is then used to investigate the temporal variability and calculate the linear trend of the areal extents. Since this method does not identify the location of the SD boundary, we use a modified endpoint method following Thomas & Nigam^2^ to delineate the boundary.

In this method, we first calculate the linear trend of a variable ($Y$) at each grid point (Eq. (6)) using a least squares fit. Then the mean value of *y*′, is corrected to preserve the original mean of variable $Y$ (Eq. (7)).

6 $$y{\prime} =kt+c,$$

7 $$y=y{\prime} +(\bar{Y}-\overline{y{\prime} }),$$

where $t\,{\rm{is}}\,{\rm{time}},k$ and $c$ are regression coefficients, and $\bar{Y}$ and $\overline{y{\prime} }$ are climatological means of $Y$ and *y*′, respectively. Equations (6–7) are applied to observed and simulated temperature, precipitation, LAI, and tree coverage at each grid point. The value *y* in Eq. (7) is used to calculate the yearly SD locations according to the climate or vegetation indices. The difference in indices between the locations in two years (endpoints) is regarded as advance/retreat regions during the two years.

Since the time series show strong multi-decadal variations in some areas, such as the SD (a substantial expansion before the 1980s and a retreat afterwards), a piecewise model is applied to detect the linear trend of a variable $Y$ with one turning point ($tp$) by using Eq. (8) and with two turning points ($t{p}_{1}$ and $t{p}_{2}$) by using Eq. (9) to replace Eq. (6), which had no turning point.

8 $$y{\prime} =\{\begin{array}{ll}{k}_{1}t+c, & t\le tp,\\ {k}_{1}t+{k}_{2}(t-tp)+c, & t > tp,\end{array}$$

9 $$y{\prime} =\{\begin{array}{ll}{k}_{1}t+c, & t\le t{p}_{1},\\ {k}_{1}t+{k}_{2}(t-t{p}_{1})+c,\, & t{p}_{1} < t\le t{p}_{2},\\ {k}_{1}t+{k}_{2}(t-t{p}_{1})+{k}_{3}(t-t{p}_{2})+c, & t > t{p}_{2}\end{array}$$

where ${k}_{1}$, ${k}_{2}$, ${k}_{3}$, and $c$ are regression coefficients and $tp$ is also to be determined through the regression process. Equations (8–9) have been widely used to detect turning points^52,53^.
